# Influence of white-light-emitting diodes on primary visual cortex layer 5 pyramidal neurons (V1L5PNs) and remodeling by blue-light-blocking lenses

**DOI:** 10.1007/s10792-024-03036-6

**Published:** 2024-02-28

**Authors:** Susmitha Mattam, R. Huban Thomas, Elizebeth O. Akansha, Judith S. Jathanna, Radhika R. Poojary, Shailaja Sarpangala, Judy Jose, Nagarajan Theruveethi

**Affiliations:** 1 Department of Optometry, Manipal College of Health Professions, Manipal Academy of Higher Education, Manipal, 576104 India; 2 Department of Anatomy, Kasturba Medical College Manipal, Manipal Academy of Higher Education, Manipal, Karnataka 576104 India; 3 Department of Ophthalmology, Kasturba Medical College Manipal, Manipal Academy of Higher Education, Manipal, Karnataka 576104 India; 4 Sankara College of Optometry, Hyderabad, 500032 India; 5 University of Houston College of Optometry, Houston, USA

**Keywords:** Light-emitting diodes, Blue-light-blocking lenses, Behavior analysis, Retinal damage, Visual cortex pyramidal neurons

## Abstract

Studies have explored the consequences of excessive exposure to white-light-emitting diodes (LEDs) in the retina. Hence, we aimed to assess the implications of such exposure on structural alterations of the visual cortex, learning and memory, and amelioration by blue-light-blocking lenses (BBLs). Eight-week-old *Wistar rats* (*n* = 24) were used for the experiment and divided into four groups (*n* = 6 in each group) as control, white LED light exposure (LE), BBL Crizal Prevencia-1 (CP), and DuraVision Blue-2 (DB). Animals in the exposure group were exposed to white LED directly for 28 days (12:12-h light/dark cycle), whereas animals in the BBL groups were exposed to similar light with BBLs attached to the LEDs. Post-exposure, a Morris water maze was performed for memory retention, followed by structural analysis of layer 5 pyramidal neurons in the visual cortex. We observed a significant difference (*P* < 0.001) in the functional test on day 1 and day 2 of training in the LE group. Structural analysis of Golgi-Cox-stained visual cortex layer 5 pyramidal neurons showed significant alterations in the apical and basal branching points (*p* < 0.001) and basal intersection points (*p* < 0.001) in the LE group. Post hoc analysis revealed significant changes between (*p* < 0.001) LE and CP and (*p* < 0.001) CP and DB groups. Constant and cumulative exposure to white LEDs presented with structural and functional alterations in the visual cortex, which are partly remodeled by BBLs.

## Introduction

Light is omnipresent and essential for quality of life. Artificial light sources, such as LEDs, might have detrimental effects on ocular health [1–3]. Visible light plays a chief role in image forming [4–7] and non-image forming functions such as sleep/wake states, alertness, mood, and behavior [8–12]. Visible light has an acute impact on cortical functioning [13–15]. Due to their efficiency and durability, LEDs have become a predominant light source in recent years [16]. These LEDs have a peak emission of blue wavelength (400–455 nm) [17, 18], which produces high energy and causes unexpected ocular alterations that could be both advantageous and disadvantageous [12, 19–21].

Cumulative light exposure causes retinal damage through photothermal, photomechanical [20], and photochemical damage [22–24], which might lead to decreased rhodopsin concentration [25], necrosis, loss of photoreceptors [26–28], increased risk of cataract, macular degeneration [29], RPE degeneration [30, 31], and retinal cell death [28] resulting in irreversible vision loss [32].

Exposure to hazardous blue wavelength causes damage of retinal pigmented epithelium (RPE), rods and cones, and the intrinsically retinal ganglion cells (ipRGCs) to create hazardous reactive oxygen species resulting in irreversible photochemical damage, leading to apoptosis [12, 33]. Damage in rods and cones leads to damage of ipRGCs, which inhibit the photic melatonin [8] and establish different synaptic connections within the retina projecting to distinct brain centers [34]. These ipRGCs play a direct role in visual luminance coding in the thalamus and visual cortex [35]. The retina is connected to the suprachiasmatic nucleus (SCN), which is the central pacemaker through the retinohypothalamic tract [9]. The optic nerve terminates on the cells of the lateral geniculate nucleus (LGN) and projects to the primary visual cortex (Striate cortex; V1), which begins to reconstitute the image from the receptive fields of the cells of the retina. Studies show that damages in retinal ganglion cells (RGCs) might alter the visual cortex neurons, altering visual functions [36, 37].

Current lens manufacturing industries claim that blue-light-blocking lenses (BBLs) protect photochemical retinal damage by absorbing the hazardous blue wavelength, attributable to their filtering properties reducing exposure to blue light, which may have an impact on circadian rhythm [38]. The different BBLs available in the market are Duravision Blue (Carl Zeiss, Oberkochen, Germany) and Crizal Prevencia (Essilor, Charenton-le-Pont, France). Research on animal models suggests that the visible spectrum of light causes a broad range of disruptive metabolomic changes [39] in the image and non-image-forming pathways. Due to the dearth of existing evidence, we studied the impact of white LED exposure on behavior and visual cortex pyramidal neuron structure and amelioration by commercially available BBLs.

## Methodology

### Ethical statement

This study was approved by the Institutional Animal Ethics Committee (IAEC/KMC/35/2020) of Kasturba Medical College, Manipal Academy of Higher Education (MAHE), Manipal. Following the approval, healthy adult male 8-week-old *Wistar rats* (*n* = 24) were procured from the Central Animal Research facility, MAHE. Animal handling and investigational procedures were carried out in accordance with the prescribed guidelines from CPCSEA (94/PO/Re Bi/5/99/CPCSEA).

### Standardization of light exposure and laboratory setup

The procured animals were housed in a controlled laboratory setup in sterile polypropylene cages (*L* = 100 cms, *W* = 70 cms, *H* = 50 cms) with paddy husk bedding, including water and a standard pellet diet available *ad-libitum* [40]. The healthy rats were randomly divided into four groups: (1) control group (NC, *n* = 6) (2), white-light exposure group (LE, *n* = 6), (3) BBL-I—(Crizal Prevencia (CP, *n* = 6)) and (4) BBL-II—(Duravision Blue (DB, *n* = 6)). Animals in the NC group were maintained under a normal laboratory environment, whereas the LE group was exposed to white LEDs (450–500 lx) directly for 28 days (12:12-h dark/light cycle) to match the nocturnal time of the rodents with 100% light output. The light properties were standardized using a spectrometer (“Asensetek Lighting Passport Pro Spectrometer | Ushio America, Inc.,”) [41]. The white LEDs were fixed at a height of 52 cm on the top of the cage with a uniform illumination of 450–500 lx uniform exposure to light was maintained throughout the cage. For the treatment groups, the BBLs (CP and DB) were fixed to the LEDs and sealed to prevent direct light exposure.

### Behavioral assessment

The Morris water maze test was conducted in an open circular pool with a 150 cm diameter and a 40 cm depth with four imaginary quadrants filled approximately halfway with water and equilibrated the water temperature to room temperature of 18–22 °C [42]. The concealed platform was immersed 1 cm below the water surface in one of the four quadrants considered the target quadrant. This hidden platform was (4″ × 4″) camouflaged with non-toxic white tempera paint, rendering it indistinguishable due to the low visual aspect ratio to the water seen by the animal while swimming [40, 43–45]. The orientation was facilitated by maintaining a definite visible cue (symbol ‘ + ’; 10 cm *H* = 10 cm, *W* = 10 cm, 100% contrast black target on white background) in the target zone [45]. The four quadrants in the pool are considered four zones, and the target zone is the fifth zone. A video camera (Logitech B525 HD Webcam) connected to a computer system (HANNS-G) was placed above the center of the pool to record the video and capture images (640 × 480 pixels). This computer system had special tracking software (ANY-maze version 4.82) to track the animals' movement and assess the recorded video [46].

### Water maze prep training

After 28 days of LED exposure, all animal groups underwent four consecutive days of training, with each day consisting of four trials. During the prep training, an animal was placed on a platform kept at the center of a pool of water at 26 °C. The platform was exposed one inch above the water's surface to make the animal aware of its presence. Each animal underwent four trials, placing it on the platform for twenty seconds. The water maze had four starting positions, exploring different directions (anterior, posterior, right and left lateral) and the animal was taken to one of these positions. To avoid accentuating the animal, it was lowered into the water tail-end first with the support of a hand rather than dunking headfirst. The animal was allowed to find the platform within 60 s. If the animal failed to find the platform within the given time, it was trained to swim to the platform by being guided gently. This training procedure was repeated until the animal learned to find the platform. This process was repeated for four trials, each starting from a different position. After completing all four trials, the animal was warmed with a cotton towel. Post-training, the water maze test was performed.

### Morris water maze testing

The rats were placed facing the pool’s sidewalls from different starting positions, and the time taken to reach the hidden platform was recorded. Following the last training session, the animals were subjected to one session of memory retention test, during which the hidden platform was removed. The time to reach the target quadrant during the four consecutive training days and the memory retention test was calculated in seconds. Each animal’s total time to reach the target quadrant was measured in seconds [47]. On the fifth day, the hidden platform was removed from sight before conducting a memory retention test (60 s), which lasted for each animal. Latency (> 60 s) reaching the target quadrant suggested memory impairment. The time taken to reach the target quadrant was compared across all the groups.

## Structural assessment

### Golgi Stain impregnation and tissue processing

Post-behavioral assessment, the animals were placed in the laboratory environment for two days before scarifying. The animals were killed with an overdose of Diethyl Ether 98% (LOBA CHEMIE PVT.LTD.), and the brain tissue was harvested and impregnated into freshly prepared Golgi-Cox stain (brain was separated into two hemispheres for better impregnation) for 21 days. Hemispheres are immersed in each tissue sample container of 10 ml Golgi-Cox solution and stored in a dark room at 24 °C. The Golgi-Cox solution was prepared 24 h prior and replaced once every five days for 21 days to ensure uniform staining and better penetration. Mercury chloride (Medilise Chemicals, KRL/KNR/00087/2003), potassium chromate, potassium dichromate, and distilled water were dissolved using a magnetic stirrer (ROTEK magnetic stirrer) to prepare Golgi-Cox solution. Post-Golgi-Cox staining (21 days of fixation), the brain tissue was fixed to the sledge microtome (Size 250 mm × 210 mm, H-325 × W-260 × D-610 mm) (Radical scientific equipment, Pvt. Ltd) plate with one drop of quick (PELCO^®^ Pro CA44 Tissue Adhesive) fix glue and sections of visual cortex with a thickness of 150 μm were obtained. The tissue sections were collected with a thick brush (Camel Hair Brushes, 3.18 mm wide) and transferred to tissue cassette (Leica-lp-c-biopsy-cassettes) and soaked in a 5% sodium carbonate solution for 20 min to enable clear visibility of tissues and neurons. The tissue sections were then dehydrated in ethyl alcohol (99.9% ethanol, UN No. 1170) in grades of 70% for 10 min (thrice), 90% for 10 min (thrice), and pure alcohol (99.9%) for 10 min (thrice) before being cleared with sulfur-free xylene (Spectrum) rendering the tissues transparent. Finally, those tissues were mounted on microscopic slides (BIOCRAFT, 26 × 76MM, CAT NO. 7101) using dibutyl phthalate Polystyrene Xylene (DPX, Sigma-Aldrich).

### Neuron imaging and quantification

Motic Images Plus 2.0 ML software was used to capture microphotographs (every 5 µm thickness), well-stained neurons were captured using a light microscope with a digital microscope camera (Moticam 580, 5.0 MP; Model no. 12000425). A total of 846 neurons (36 neurons each animal) were selected from layer five pyramidal visual cortex neurons (V1L5PNs) in each group and 30 images were obtained from each neuron in the *Z*-axis using microscopy. The neurons were selected based on the following parameters: complete staining of an individual neuron, background staining, uniformity of neuronal staining and clarity in staining with dendritic spines. Any artifacts in staining were not taken into consideration during quantification. To minimize bias, two investigators (EOA and MS) manually traced dendrites from the coded slides, and the mean values were tabulated by a third investigator (RP). The neurons were traced from the soma (cell body) to assess apical and basal arborization for every 0 µm up to 140 µm and intersections from 20 up to 140 µm (5 µm precision). To obtain a precise measurement of dendrite quantification and branching, while avoiding any potential errors due to missed pruning or arborization, we took 30 images (each with a 5 µm resolution) for every neuron. These images were captured up to 150 µm from the soma (*Z* plane). This method has been demonstrated to be highly effective in minimizing errors, and the analysis technique was adopted [48, 49].

### Statistical analysis

Functional and structural changes of the visual cortex post-white LED exposure were analyzed using an R programming software environment for statistical computing (version 3.6.3, Massachusetts Institute of Technology (MIT), Cambridge, MA, USA). The data obtained after performing the MWM test (training and retention) and structural data for neuronal quantification were analyzed across all the groups using two-way analysis of variance (Two-way ANOVA) followed by Tukey’s post hoc analysis to report the significance between the groups, if any. To mitigate any potential biases, two investigators (EOA and MS) manually traced dendrites from the coded slides. The mean values were then tabulated by a third investigator (RP), and statistical analysis was carried out by SBG.

## Results

### Behavioral analysis

**Fig. 1:**
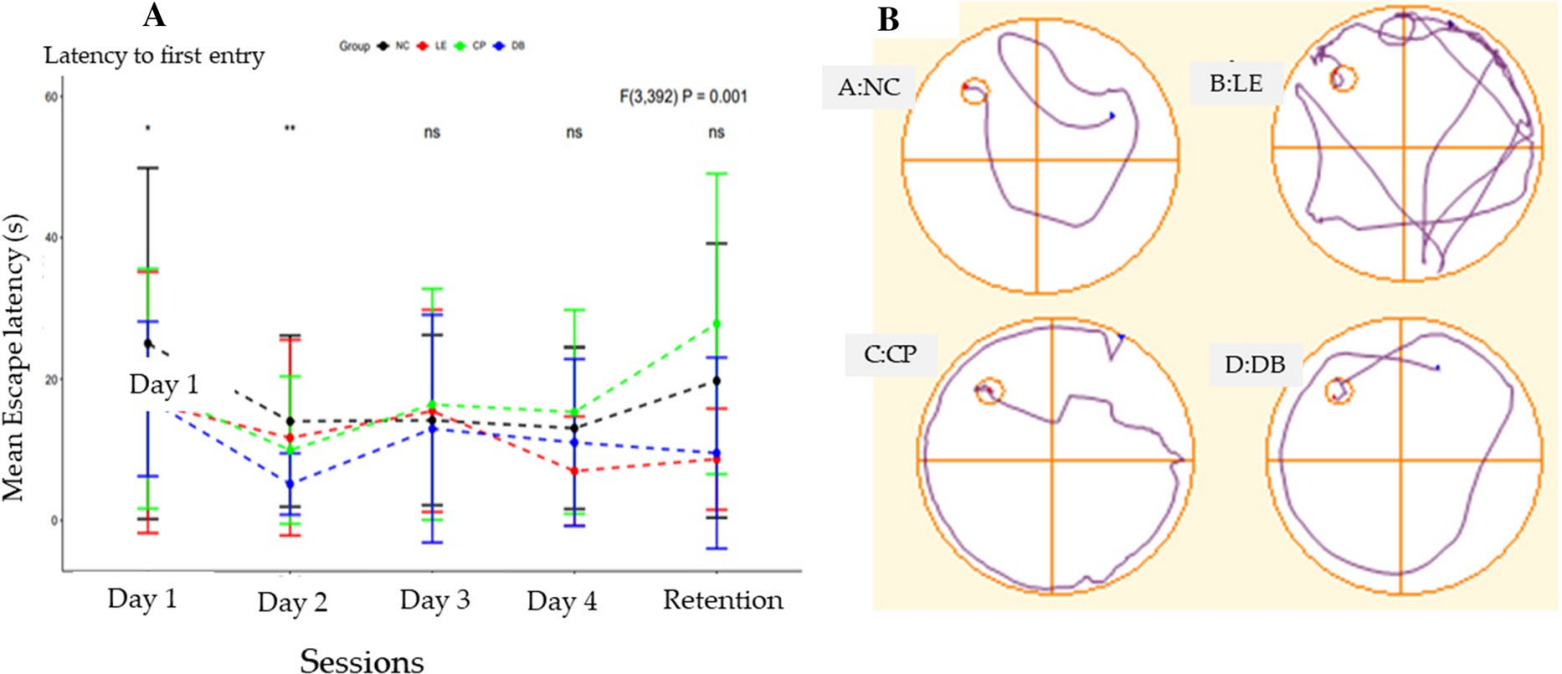
**A** The average time it took to locate the hidden platform in seconds for each trial day, as well as the memory retention test, across all animal groups. The *Y*-axis represents the mean latency in seconds, and the *X*-axis training session and retention of different days. **B** The track plot depicts the pathways taken by the animals to discover the hidden platform for all four groups, namely normal controls (NC), white LED light exposure (LE), LE+Crizal prevention (CP), and LE+Duravision blue (DB)

Post 28 days of white LED exposure, the mean latencies (in seconds) to reach the hidden platform were compared using two-way ANOVA across all the groups for all four consecutive trials and memory retention test (Fig. 1A). Two-way ANOVA revealed a statistically significant difference (*F*_3, 392_ = 0.001, *P* < 0.001). Post hoc analysis between the groups showed significance only on day 1 (*p* < 0.05) and day 2 (*p* < 0.01) of the trial, respectively. On day 3 and day 4, the animals in all the groups demonstrated a similar latency trend.

The track plots (Fig. 1B) represent the path taken by the animals in the circular pool to reach the hidden platform. Longer path lengths taken by the animals in the light exposure group represent detrimental effects on spatial learning and memory.

The average time it took to find the hidden platform over four consecutive days was 16.38 s for the control group (Fig. 1B-a), 12.57 s for the light exposure group (Fig 1B-b), 15.04 s for the CP group (Fig. 1B-c), and 11.52 s for the DB group (Fig. 1B-d). On the memory retention day, the control group took an average of 19.76 s to find the platform, while the light exposure group took only 8.6 s, the CP group took 15.67 s, and the DB group took 9.53 s.

### Structural assessment using Sholl’s grading

**Fig. 2:**
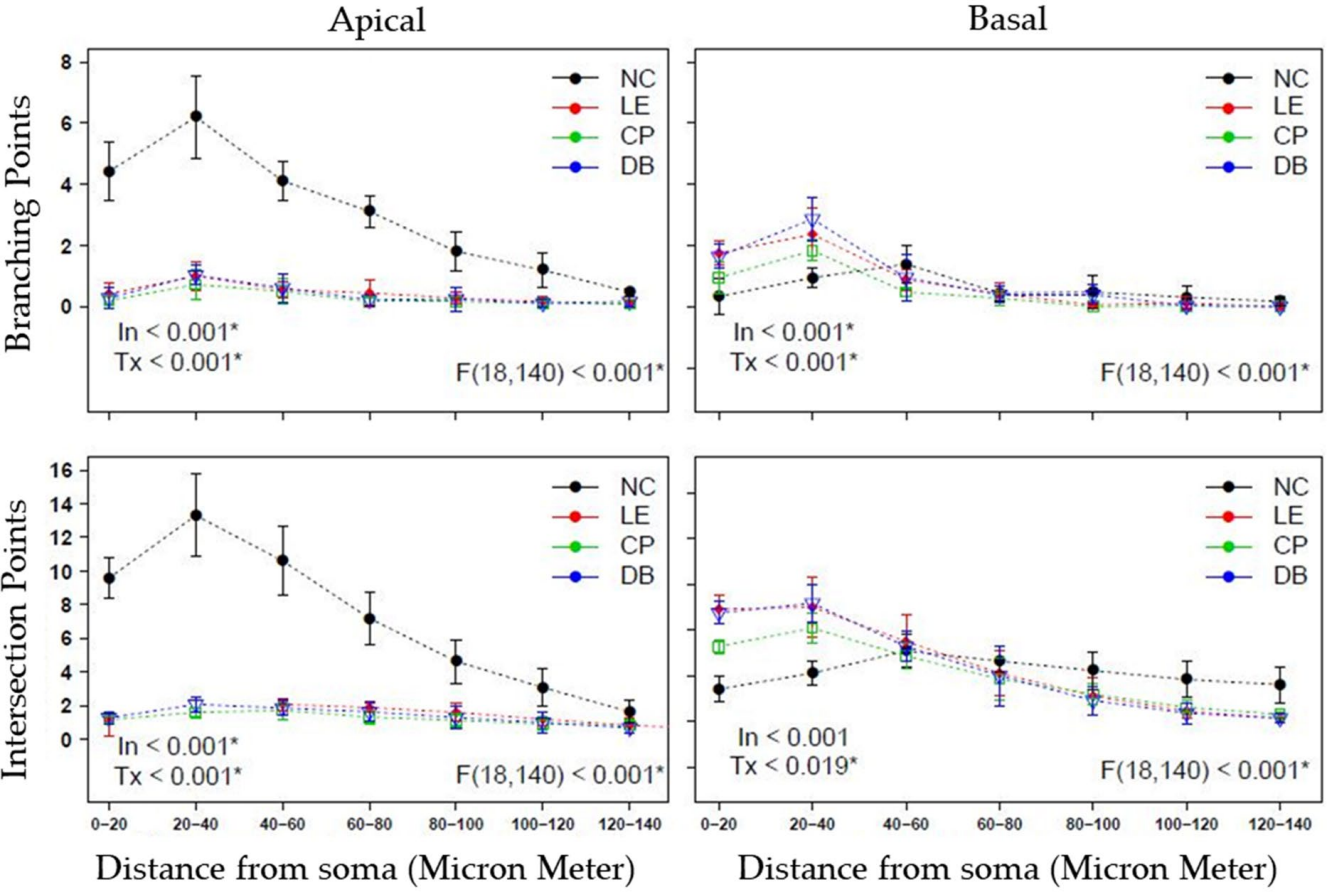
Representation of the apical and basal branching and intersection points of pyramidal neurons across all the groups, with a range of 20–140 µm. The values are presented in the Mean ± SEM, with the dots indicating the mean values and the bars indicating the standard error of the mean (SEM). The significance using Tukey’s post hoc analysis is denoted by in* for *p*<0.05. The left top corner represents apical branching, while the right top corner represents Basal branching. On the other hand, the left bottom corner represents the apical intersection, and the right bottom corner represents the Basal intersection

**Fig. 3:**
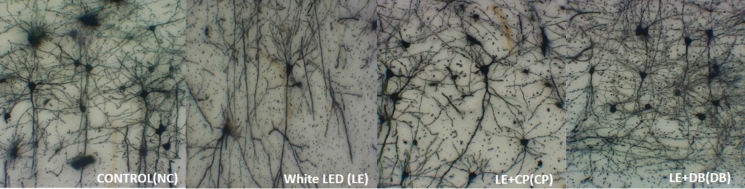
Representative photomicrograph illustration of Golgi-Cox-stained primary visual cortex layer 5 pyramidal neurons

Apical and basal branching points and intersections of Golgi-stained V1L5PNs were compared across all the groups. There was a significant reduction in apical branching and intersection points at all the distances from the soma in the LE group and BBL groups compared to the NC group with a statistically significant difference (*F*_18, 140_ = 0.001, *p* < 0.001) (Fig. 2). A similar trend was seen in basal branching points and intersections with significance (*F*_18, 140_ = 0.019, *p* < 0.001) when other groups showed reduced basal branching and intersection points. This illustrates that the DB group had more branching points than the NC, LE, and CP groups (Fig. 3). These findings showed that white LED exposure has caused degenerative alterations in the neurons of the visual cortex region.

## Discussion

Our study found that after 28 days of exposure to white LEDs, the animals in the LE group demonstrated impaired spatial learning and memory, indicated by behavioral assessment. Structural assessment of Golgi-stained V1L5PNs demonstrated neurodegeneration by shortening of apical and basal dendrites of V1L5PNs at all distances from the soma. BBLs (CP and DB) demonstrated partial protective efficacy functionally by improving spatial learning, memory, and neuronal structure.

Chronic exposure to white light can lead to alterations in the regulation of circadian rhythm, melatonin suppression, hormone secretion, mood swings, and behavior [8, 9, 22, 50–53]. Cells possess coping mechanisms such as surviving enormous stressful periods, adapting to chronic stress, remodeling the physiological demands dependent on the various stressful conditions, oxidative stress, aging, increased biosynthesis, inflammation, and protein misfolding [54–56]. Studies show that damages in RGCs might alter the visual cortex neuron, and these damages potentially alter the image and non-image-forming vision [36, 37]. Due to the lack of existing evidence illustrating the effect of light exposure, we demonstrated the effect of cumulative white LED exposure on the visual cortex neurons and amelioration by BBLs.

However, our study presents some limitations. The exposure period was shorter (28 days) and lacked retinal structural assessment, which could provide insight into the pathway leading to visual cortex neuronal degeneration. For future investigation, damage in the retinal layers can be assessed with a longer exposure period (90 days).

## Conclusion

Prolonged and consistent exposure to white LED lights on a 12:12 light/dark cycle led to notable behavior alterations with impaired spatial learning. This was particularly evident in the light exposure group, compared to other groups, which showed retrograde pruning of layer 5 pyramidal neurons of the visual cortex. The blue-blocking lenses can extend trivial protection against white LED exposure. To gain a deeper understanding of our findings, it would be beneficial to explore the Intracellular cortical signaling pathway in the context of retrograde degeneration. This would provide valuable insights into the underlying mechanisms at play.

## Data Availability

The data presented in this study are available on request from the corresponding author.
